# Preparation of goat and rabbit anti-camel immunoglobulin G whole molecule labeled with horseradish peroxidase

**DOI:** 10.14202/vetworld.2017.92-100

**Published:** 2017-01-23

**Authors:** Eman Hussein Abdel-Rahman, Jakeen Kamal El-Jakee, Mahmoud Essam Hatem, Nagwa Sayed Ata, Ehab Ali Fouad

**Affiliations:** 1 Department of Parasitology and Animal Diseases, National Research Centre, Egypt; 2 Department of Microbiology, Faculty of Veterinary Medicine, Cairo University, Egypt; 3 Department of Microbiology and Immunology, National Research Centre, Egypt

**Keywords:** anti-camel immunoglobulin G, *Camelus dromedarius*, conjugation, horseradish peroxidase, purification

## Abstract

**Aim:**

As the labeled anti-camel immunoglobulins (Igs) with enzymes for enzyme-linked immunosorbent assay (ELISA) are unavailable in the Egyptian market, the present investigation was directed for developing local labeled anti-camel IgG with horseradish peroxidase (HRP) to save hard curacy.

**Materials and Methods:**

For purification of camel IgG whole molecule, camel sera was preliminary precipitated with 50% saturated ammonium sulfate and dialyzed against 15 mM phosphate-buffered saline pH 7.2 then concentrated. This preparation was further purified by protein A sepharose affinity column chromatography. The purity of the eluted camel IgG was tested by sodium dodecyl sulfate polyacrylamide gel electrophoresi. Anti-camel IgG was prepared by immunization of goats and rabbits separately, with purified camel IgG. The anti-camel IgG was purified by protein A sepharose affinity column chromatography. Whole molecule anti-camel IgG was conjugated with HRP using glutraldehyde based assay. Sensitivity and specificity of prepared conjugated secondary antibodies were detected using positive and negative camel serum samples reacted with different antigens in ELISA, respectively. The potency of prepared conjugated antibodies was evaluated compared with protein A HRP. The stability of the conjugate at −20°C during 1 year was assessed by ELISA.

**Results:**

The electrophoretic profile of camel IgG showed four bands of molecular weight 63, 52, 40 and 33 kDa. The recorded sensitivity and specificity of the product are 100%. Its potency is also 100% compared to 58-75% of commercial protein A HRP. The conjugates are stable for 1 year at −20°C as proved by ELISA.

**Conclusion:**

Collectively, this study introduces goat and rabbit anti-camel IgG whole molecules with simple, inexpensive method, with 100% sensitivity, 100% specificity and stability up to 1 year at −20°C. The important facet of the current study is saving hard curacy. Future investigations are necessary for preparation of IgG subclasses.

## Introduction

The camel occupies a unique position among animals which man has failed to exploit adequately and undoubtedly have unexplored and unrealized potential. From a global perspective, little interests are directed to the economic significance of camel production in comparison with that of other domestic animals. However, camels breeding could be participated efficiently in providing Egyptian’s needs of animal protein and its by-products. The main breed is the dromedary of the Nile Delta of Egypt [1]. Improvement of camel production depends on efficient management and diseases control which in turn through understanding of the functioning of its various biological systems, such as the immune system [2]. Concerning camel disease, camels were formerly considered resistant to most of the diseases commonly affecting livestock, but as more research was conducted; camels were found to be susceptible to a large number of pathogenic agents [3].

The availability of proper serodiagnostic aids help in diagnosing a wide range of infectious diseases infects camels [4]. The different serologic tests that were used to diagnose viral, bacterial, and parasitic infections depend on antigen-antibody reactions such as enzyme-linked immunosorbent assay (ELISA) [5,6]. This test needs enzymes conjugated secondary antibodies that are lacked in the Egyptian market and need to be imported with hard curacy.

Hence, the ultimate aim of this study is participating in solving the problem of camel diagnostic reagents lack through introduce one-way to monitor the camel immune responses to different pathogens by developing conjugated secondary anti-camel IgG with horseradish peroxidase (HRP). This participation will facilitate accurate diagnosis and control of infectious diseases, which could affect camel welfare and productivity and save hard curacy.

## Materials and Methods

### Ethical approval

Experiments were performed according to the Guide for the care and use of Laboratory animals and Ethical Approval of Animal Rights according to Committee, National Research Centre, Egypt.

### Materials

Protein A sepharose gel has a high binding capacity toward camel immunoglobulin G (IgG). Ammonium sulfate (NH_4_)_2_SO_4_, monobasic sodium phosphate (NaH_2_PO_4_), dibasic sodium phosphate (Na_2_HPO_4_), orthophenylenediamine, HRP, bovine serum albumin, and protein A conjugate peroxidase were purchased from Sigma. Sera from camels, immunized goats and rabbits were used as the source of polyclonal camel IgG and anti-camel IgG (Bacterial and parasitic antigens were donated by Prof. Dr. Eman H. Abdel-Rahman).

### Methods

#### Precipitation of Igs with ammonium sulfate solution

About 50% saturated ammonium sulfate solution (SAS) was used to precipitate Igs. Ammonium sulfate was removed by dialysis against 15 mM phosphate-buffered saline (PBS) for 3 days at 4°C. The Igs were concentrated by polyethylene glycol or lyophilization according to Abd El Hafez *et al*. [7].

#### Purification of IgGs by protein A affinity chromatography

Protein A sepharose gel was used to purify IgG using eluting buffer 0.1 M glycine. The separation method was according to Abd El Hafez *et al*. [7].

#### Estimation of protein contents of Igs

The assay was performed according to the method of Lowry *et al*. [8].

#### Electrophoresis of camel IgG in sodium dodecyl sulfate polyacrylamide gel electrophoresi (SDS-PAGE)

SDS-PAGE was performed in 10% polyacrylamide gels according to Laemmli [9]. Camel IgG was mixed with sample buffer containing 2-mercaptoethanol before loading to the gel. After separation, slab gel was stained with Coomassie Brilliant Blue dye. Relative molecular weights of bands were calculated using Protein marker supplied by Sigma-Aldrich. Molecular weights were determined using Bio Rad Gel Doc XR+ Apparatus.

### Preparation of anti-camel IgG in goats and rabbits

Animals immunization was performed according to Muro *et al*. [10]. The camel IgG was used in concentration of 40 µg/kg body weight of animal and was thoroughly mixed with an equal volume of complete Freund’s adjuvant (Difico, USA) and injected subcutaneously. 2 weeks later, first booster dose was given by the same way but with incomplete Freund’s adjuvant. The second and third booster doses injected after 21 and 28 days, respectively, without adjuvant. ELISA adopted to evaluate anti-camel IgG production in both goats and rabbits. Antigen concentration, antibody dilution, and anti-species secondary conjugated antibody were determined with checkerboard titration and the assay performed according to Paul and Akira [11].

### Labeling of rabbit and goat IgGs with HRP

About 10 mg of HRP mixed with 5 mg of IgG in 1 ml total volume of 100 mM phosphate buffer (pH 6.8). The mixture was dialyzed over night at 4°C against 100 mM phosphate buffer (pH 6.8). 50 µl of the diluted glutaraldehyde added to the dialyzed mixture with gentle stirring at room temperature for 3 h. 2 M glycine solution added to obtain a 0.1 M final concentration. The mixture was left at room temperature for 2 h and then dialyzed over night at 4°C against 100 mM PBS. It centrifuged for 30 min at 10,000 ×*g* at 4°C. The supernatant transferred in a suitable tube and tone volume of distilled glycerol added. The conjugate stored at −20°C. The procedures performed according to the method of Avrameas [12].

### Assessment of prepared conjugates potency compared with commercial protein A conjugate

ELISA used to assess the development of anti-camel IgG in goats and rabbits and to determine the optimum dilutions of prepared conjugates, antigens, and sera concentrations. It was also used to evaluate the potency of prepared conjugates compared with protein A peroxidase and its stability. The assay performed according to Paul and Akira [11].

## Results

Protein content of purified camels, goats and rabbits IgG are shown in Table 1

**Table-1:** Protein content of purified camels, goats, and rabbits IgG.

Animal	Protein content of Igs mg/ml	Protein content of IgG mg/ml
Camel	3.5	2.3
Goat	2.3	1.96
Rabbit	2.7	2.1

IgG: Immunoglobulin G

### SDS-PAGE profile of camel IgG

**Figure-1:**
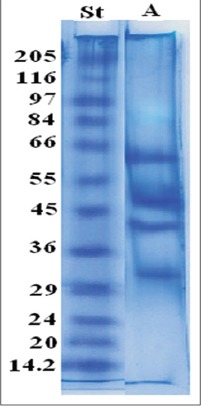
Sodium dodecyl sulfate polyacrylamide gel electrophoresi profile of camel immunoglobulin G. Lane St: Molecular weight marker, Lane A: Camel immunoglobulin G.

The electrophoretic profile of camel IgG showed four bands of molecular weight 63, 52, 40 and 33 kDa (Figure-1).

### Development of anti-camel IgG in goats and rabbits

**Figure-2:**
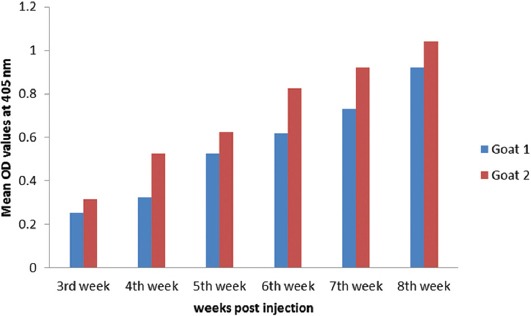
Time based goats antibody response to camel immunoglobulin G measured by enzyme-linked immunosorbent assay.

**Figure-3:**
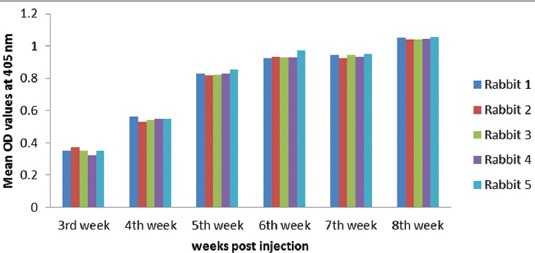
Time based rabbits antibody response to camel immunoglobulin G measured by enzyme-linked immunosorbent assay.

As proved by ELISA, the appropriate camel IgG concentration is 20 µg/ml, goat and rabbit antibody dilution is 1:100 and anti-species secondary conjugated antibody is 1:1000 based on checkerboard titration. Time based goats and rabbits antibody response to camel IgG as shown in Figures-2 and 3.

### Potency of prepared goat and rabbit anti-camel HRP in ELISA

**Figure-4:**
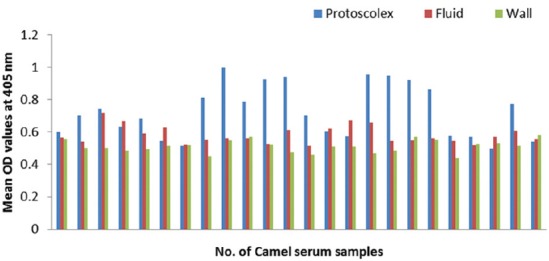
Comparative evaluation of goat anti-camel horseradish peroxidase potentials in detection of immunoglobulin G in camel serum samples react with different *Echinococcus granulosus* antigens.

**Figure-5:**
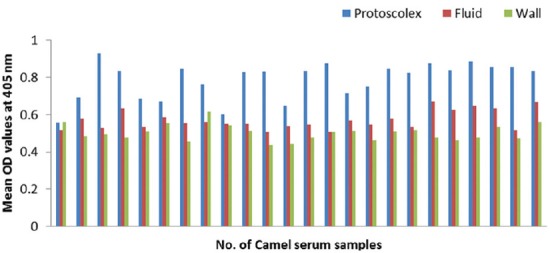
Comparative evaluation of rabbit anti-camel horseradish peroxidase potentials in detection of immunoglobulin G in camel serum samples react with different *Echinococcus granulosus* antigens.

**Figure-6:**
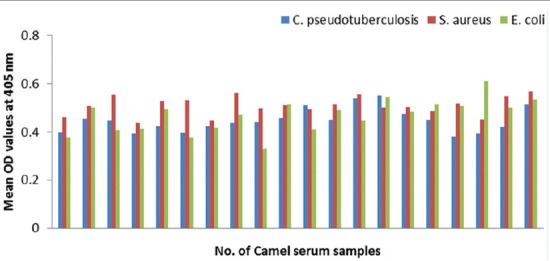
Comparative evaluation of goat anti-camel horseradish peroxidase potentials in detection of immunoglobulin G in camel serum samples react with different bacterial antigens.

**Figure-7:**
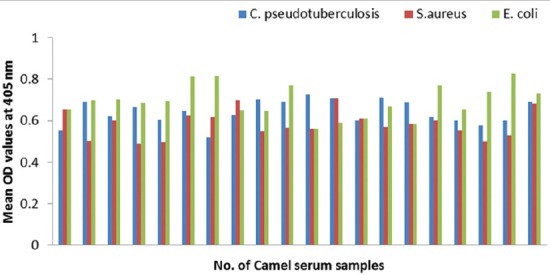
Comparative evaluation of rabbit anti-camel horseradish peroxidase potentials in detection of immunoglobulin G in camel serum samples react with different bacterial antigens.

The optical density (OD) values recorded due to reaction of diseased camel serum samples with different antigens of *Echinococcus granulosus*; wall, protoscolex and fluid antigens as well as bacterial antigens prepared from *Corynebacterium pseudotuberculosis, Staphylococcus aureus*, and *Escherichia coli* using prepared conjugates in ELISA proved validity of both conjugates as shown in Figures-4, 5, 6 and 7.

#### Conjugates sensitivity

**Figure-8:**
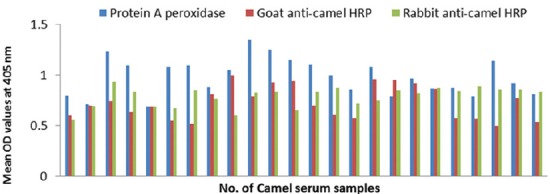
Comparative sensitivities of protein A peroxidase, goat and rabbit anti-camel horseradish peroxidase in detection of immunoglobulin G in positive camel serum samples specific to *Echinococcus granulosus* protoscolex antigen.

**Figure-9:**
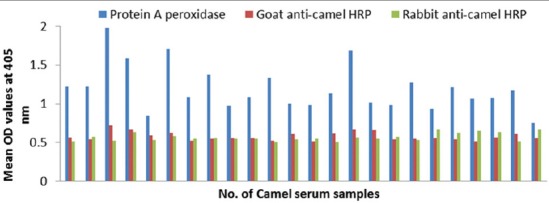
Sensitivity evaluation of goat and rabbit anti-camel horseradish peroxidase compared with protein A peroxidase in detection of immunoglobulin G in positive camel serum samples reacted with *Echinococcus granulosus* fluid antigen.

**Figure-10:**
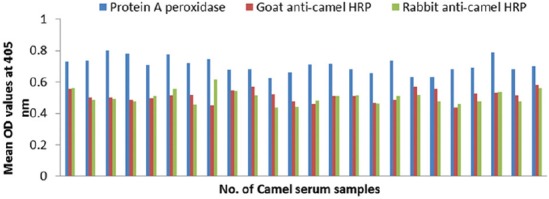
Sensitivity evaluation of goat and rabbit anti-camel horseradish peroxidase compared to protein A peroxidase in detection of immunoglobulin G in positive camel serum samples reacted with *Echinococcus granulosus* wall antigen.

The sensitivity of prepared conjugates is 100% (Figures-8, 9 and 10).

#### Conjugates specificity

**Figure-11:**
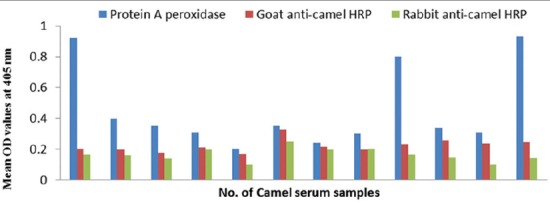
Specificity evaluation of goat and rabbit anti-camel horseradish peroxidase compared with protein A peroxidase using negative camel serum samples and *Echinococcus granulosus* protoscolex antigen.

**Figure-12:**
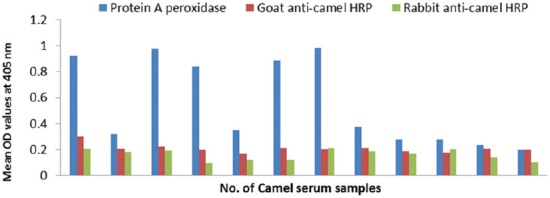
Specificity evaluation of goat and rabbit anti-camel horseradish peroxidase compared with protein A peroxidase using negative camel serum samples and *Echinococcus granulosus* fluid antigen.

**Figure-13:**
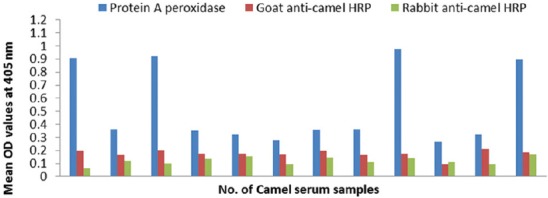
Specificity evaluation of goat and rabbit anti-camel horseradish peroxidase compared with protein A peroxidase using negative camel serum samples and *Echinococcus granulosus* wall antigen.

The specificity of prepared conjugates is 100 % compared to 58-75% of commercial protein A HRP that are shown in Table 2 and Figures-11, 12 and 13).

**Table-2:** Comparitive specificities of protein A peroxidase, goat and rabbit anti-camel HRP using negative camel serum samples and *E. granulosus* antigens.

Number of camel samples	OD values of−ve camel serum samples and *E. granulosus* antigens								

	Protoscolex antigen			Fluid antigen			Wall antigen		
		
	Protein A peroxidase	Goat anti- camel HRP	Rabbit anti-camel HRP	Protein A peroxidase	Goat anti- camel HRP	Rabbit anti-camel HRP	Protein A peroxidase	Goat anti-camel HRP	Rabbit anti-camel HRP
1	0.925*	0.201	0.165	0.921*	0.301	0.210	0.905*	0.198	0.066
2	0.399	0.199	0.158	0.319	0.210	0.185	0.362	0.165	0.122
3	0.352	0.175	0.140	0.978*	0.221	0.194	0.921*	0.201	0.101
4	0.311	0.210	0.197	0.841*	0.196	0.098	0.354	0.175	0.135
5	0.202	0.166	0.099	0.351	0.165	0.124	0.324	0.177	0.157
6	0.352	0.325	0.249	0.886*	0.214	0.123	0.275	0.169	0.096
7	0.242	0.215	0.199	0.984*	0.201	0.214	0.356	0.197	0.145
8	0.300	0.199	0.201	0.378	0.211	0.187	0.365	0.165	0.111
9	0.798*	0.231	0.165	0.279	0.189	0.165	0.977*	0.175	0.143
10	0.341	0.258	0.147	0.281	0.177	0.203	0.265	0.098	0.109
11	0.309	0.236	0.098	0.234	0.206	0.144	0.321	0.210	0.097
12	0.932*	0.245	0.142	0.198	0.198	0.102	0.898*	0.187	0.169

Standard deviation ranged (0.007-0.01),

* Positive. HRP=Horseradish peroxidase, OD=Optical density, *E. granulosus*=*Echinococcus granulosus*

#### Conjugates stability

**Figure-14:**
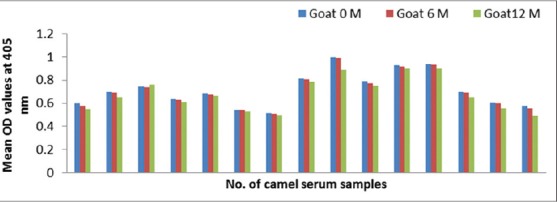
Significant stability of stored goat anti-camel horseradish peroxidase for 1 year at −20°C.

**Figure-15:**
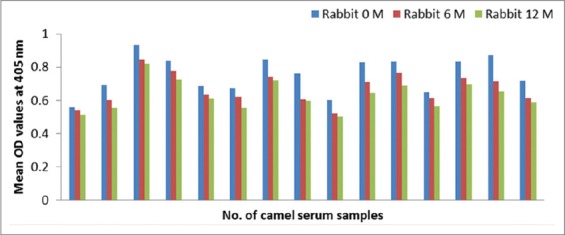
Significant stability of stored rabbit anti-camel horseradish peroxidase for 1 year at −20°C.

The conjugates are stable for 1 year at −20ºC that are shown in Table 3 and (Figures-14 and 15).

**Table-3:** Stability of stored conjugates at −20°C for 1 year.

Number of camel serum sample	Mean OD values±standard deviation					

	Goat anti-camel HRP			Rabbit anti-camel HRP		
	
	Freshly prepared	Stored for 6 months at −20°C	Stored for 1 year at −20°C	Freshly prepared	Stored for 6 months at −20°C	Stored for 1 year at −20°C
1	0.600	0.578	0.550	0.557	0.540	0.514
2	0.701	0.695	0.655	0.693	0.600	0.554
3	0.742	0.741	0.761	0.932	0.845	0.822
4	0.635	0.629	0.614	0.836	0.777	0.725
5	0.684	0.677	0.666	0.686	0.632	0.610
6	0.546	0.546	0.532	0.672	0.620	0.554
7	0.514	0.509	0.500	0.847	0.742	0.721
8	0.811	0.805	0.785	0.763	0.604	0.598
9	0.997	0.990	0.888	0.600	0.521	0.500
10	0.788	0.772	0.750	0.829	0.711	0.644
11	0.925	0.914	0.899	0.832	0.765	0.689
12	0.940	0.934	0.900	0.650	0.612	0.565
13	0.701	0.695	0.650	0.834	0.735	0.699
14	0.605	0.600	0.555	0.875	0.714	0.654
15	0.575	0.555	0.489	0.716	0.612	0.588

HRP=Horseradish peroxidase, OD=Optical density

## Discussion

This study directed for development of polyvalent anti-camel IgG labeled with HRP. The procedure followed in this study was precipitation of camel Igs by SAS. The selection of this method was based on the previous results demonstrated that ammonium sulfate gives the best combination of high purity and yield, also ammonium sulfate is inexpensive and used for purification of Igs in large scale production [13,14]. The best condition for fractionation of IgG from the other proteins in camel serum was use of 55% ammonium sulfate which is comparable to the condition used in the current study proved by Khamehchian *et al*. [15].

The purification of IgG using protein A Sepharose affinity chromatography was used in the present study, to isolate IgG from camels, goats and rabbits serum samples. Adoption of this method is easy and gives large amount of highly purified yield of IgG [16]. This approach was supported with Jungbauer *et al*. [17] who compared affinity chromatography with protein A sepharose and protein G Sepharose methods for purification. Protein A sepharose was shown to be a powerful method for the isolation and purification of IgG antibodies from camels serum. While affinity chromatography with protein G sepharose bonded weakly to camel’s IgG and the performed experiment showed high losses of IgG which could be unacceptable.

Moreover, purification of serum IgG by affinity chromatography is more efficient than with diethylaminoethy-cellulose column chromatography that is more expensive. The affinity chromatography is simple, one step and highly efficient method for IgG purification compared with ion exchange chromatography recorded by Mariam *et al*. [14] and El-Hewairy *et al*. [18]. Hence, the affinity chromatography method was used as method of choice in the present work.

If a highly purified product is required, an assay for assessing the degree of protein purity is essential. The method of choice for determining purity in the current study is SDS-PAGE. Molecular weights of camel IgG four bands were 63, 52, 40 and 33 kDa. Previously, characterization of IgG was done using 12% SDS-PAGE under reducing conditions. Protein bands were visualized after staining with Coomassie Brilliant Blue, showing four bands; two bands at 50 and 30 kDa in case of IgG_1_ while IgG_2_ and IgG_3_ produce only one band at 46 and 43 kDa, respectively [16,19,20]. Under reducing conditions, IgG_1_ was resolved into light chain (22.7 kDa) and one heavy chain (49.4 kDa). The IgG_3_ preparation was reduced to one dominant heavy-chain species of 42.1 kDa. The IgG_2_ preparation was reduced to one dominant heavy-chain species of 40.9 kDa [21]. While, using 12% SDS-PAGE to study the camel IgG that appeared as three bands of 52, 46 and 30 kDa [7]. The camel IgG as 2 bands with molecular masses of 150 and 75 kDa presented by Khamehchian *et al*. [15]. The difference in number of bands between the current results and previous ones probably attributed to differences in sample preparation, which includes breakage of disulfide bonds of the molecule using β mercaptoethanol, and consequently, determine the number of bands in addition to different used stains, silver or Coomassie to visualize separated bands.

After purification of camel IgG, goats and rabbits separately inoculated to produce anti-camel IgG. Goats and rabbits are widely used for production of specific antibodies [22]. In this study, goats and rabbits received at least four subcutaneous injections each of 40 µg/kg body weight of animal using complete and incomplete Freund’s adjuvant (Difico, USA). This approach was previously adopted by Toaleb *et al*. [23], Abdel-Rahman *et al*. [24] who administrated 40 µg/kg of rabbit using Freund’s complete and incomplete adjuvant and injected subcutaneously. Different protocols previously adopted for development of anti-camel IgG. The previous studies [22,25] injected higher concentrations of camel IgG than that in the current study and used intramuscular route. Subcutaneous route is better than intramuscular one as no ulcerative lesions appear and is less painful [16].

In this study, the success of developing anti-camel IgG in both goats and rabbits monitored by indirect ELISA in which commercial anti-goat and anti-rabbit HRP were used. Previously, agar gel diffusion test was the test of choice [7,18]. This test checked if the antibodies were developed or not and did not give any indication about the titer of developed antibodies. In contrary, ELISA not only proved the production of antibodies but also facilitated the follow-up of the titer during different intervals post injection to determine the time of blood collection accurately. The method, which was selected in the current study for preparation of goats and rabbits anti-camel antibodies, gave high IgG response to lower concentration of IgG than other techniques.

In the present work, the conjugation process was based on using glutaraldehyde as the coupling agent that resulted in conjugates capable of detecting antigen-antibody reaction. The selection of this method was based on the previous results demonstrated that glutaraldehyde was the most effective and suitable reagent for producing protein-enzyme complexes which retained a part of their enzymatic and immunological specificity [12]. The conjugation method in which glutaraldehyde is adopted produces mono-functional linkers [26]. In the present work, the conjugation process was applied using HRP as it was inexpensive, easy handling and available in the market without restrictions.

For evaluation of the validity of prepared conjugates, its sensitivity in detection of antigen-antibodies reaction was assessed. All parasitologically or bacteriologically positive camel serum samples were proved to be serologically positive as judged by ELISA in which prepared conjugates were utilized. This means that labeled conjugates could detect positive infection serologically recording 100% sensitivity. These results were compared with that obtained using commercial protein A peroxidase as reference. This comparative evaluation confirmed prepared conjugates validity where protein A peroxidase recorded also 100% sensitivity. This percentage was previously reported by El-Hewairy *et al*. [18] who prepared goat and rabbit anti-camel IgG alkaline phosphatase and recorded 100% sensitivity. While, 94.8% sensitivity of the prepared goat anti-camel IgG conjugate was recorded by El-Hewairy and Syame [27] compared with 72.2% of protein A alkaline phosphatase. This low sensitivity may be concerned with low titer of antibodies in the misdiagnosed samples or inefficient coupling process between antibody and enzyme but in all cases, it supports the success of the current trial.

In the current study, the specificity of developed conjugates was 100%. Unexpectedly, protein A peroxidase reacted positively with some negative samples recording non-specific reaction ranged from 25% to 41%. This observation could be attributed to non-specific reaction of protein A with IgGs [28].

Non-significant differences were observed in mean OD values after 1 year of conjugates storage at −20°C as proved by ELISA. In previous study, the activity of stored conjugates decreased with time at 4°C and completely lost after 1 year at −20°C [18]. This observation probably reflects the unsuccessful of conjugation process that may result in damage or breakage the bond between primary and secondary antibodies or loss in enzyme activity with its substrate or both due to storage at 4°C or −20°C.

Collectively, the current product was proved to possess high potentials as compared with protein A peroxidase and showed stable activity for 1 year at −20°C. Additional evaluation of conjugates stability needed at 4°C and for more than 1 year.

## Conclusion

From the current results, it concluded that new preparations of anti-camel IgG developed in goats and rabbits. Prepared conjugates characterized by 100% sensitivity in ELISA using positive camel serum samples. They also showed 100% specificity compared to 58-75% of protein A peroxidase using negative samples. The conjugates are stable for 1 year at −20°C. Collectively, conjugated anti-camel IgGs developed by easy, simple and inexpensive method and participate in solving the problem of camel diagnostic reagents lack in the Egyptian market in addition to save hard curacy.

## Authors’ Contributions

EHA participated in antigens preparations, conjugations and ELISA. She also participated in revising manuscripts. EAF is mainly participating for the practical part and writing the manuscript. NSA revised the results and manuscript. JKE revised the results and manuscript. MEH was sick and died before participating in research.
